# Virulence evolution in response to vaccination: The case of malaria

**DOI:** 10.1016/j.vaccine.2008.04.012

**Published:** 2008-07-18

**Authors:** M.J. Mackinnon, S. Gandon, A.F. Read

**Affiliations:** a Department of Pathology, University of Cambridge, Tennis Court Road, Cambridge CB2 1QP, UK; b KEMRI-Wellcome Trust Collaborative Research Programme, PO Box 230, Kilifi 80108, Kenya; c Génétique et Evolution des Maladies Infectieuses, Institut de Recherche pour le Développement, 911 Avenue Agropolis, 34394 Montpellier Cedex 5, France; d Centre d’Ecologie Fonctionnelle et Evolutive, 1919 route de Mende, 34293 Montpellier Cedex 5, France; e Centre for Infectious Disease Dynamics, Departments of Biology and Entomology, 208 Mueller Laboratory, The Pennsylvania State University, University Park, PA 16802, USA; f School of Biological Sciences, University of Edinburgh, West Mains Road, Edinburgh EH9 3JT, UK

**Keywords:** Virulence, Evolution, Malaria, Parasite, Trade-off hypothesis

## Abstract

One theory of why some pathogens are virulent (i.e., they damage their host) is that they need to extract resources from their host in order to compete for transmission to new hosts, and this resource extraction can damage the host. Here we describe our studies in malaria that test and support this idea. We go on to show that host immunity can exacerbate selection for virulence and therefore that vaccines that reduce pathogen replication may select for more virulent pathogens, eroding the benefits of vaccination and putting the unvaccinated at greater risk. We suggest that in disease contexts where wild-type parasites can be transmitted through vaccinated hosts, evolutionary outcomes need to be considered.

## 1 An evolutionary hypothesis for pathogen virulence

Why are pathogens virulent?^1^ Why would they run the risk of killing their host when, in doing so, they lose their ongoing source of transmission to new hosts? Some evolutionary biologists believe that the answer to this question will make it possible to design vaccines and other control measures that, in the event of eradication being impossible, drive the pathogen towards lower virulence [1], [2].

One answer to this question is that virulence is a mistake by the pathogen—an ultimately maladaptative outcome that occasionally happens when a pathogen accidentally ends up in an abnormal host environment, or when a virulent mutant has a transient competitive advantage within a host (‘short-sighted, or dead-end evolution’) [3]. An alternative (but not mutually exclusive [4]) idea is that the level of pathogen virulence observed in nature is a well-adapted outcome of both positive and negative selective forces acting on virulence. Under this hypothesis, it is reckoned that to balance the fitness cost to the pathogen of host death, there must also be a virulence-related advantage to the pathogen's fitness. This is the so-called ‘virulence trade-off hypothesis’.

Of all the explanations for virulence, the trade-off hypothesis has received most attention and a large body of theory has been derived from it. Yet it is poorly supported by data. Here we describe our studies in malaria parasites, the causative agents of a disease of global importance, in which we have comprehensively explored the trade-off hypothesis. We begin by summarising our experimental tests in a laboratory mouse-malaria system of the assumptions underlying the trade-off theory. We then ask whether the rodent data are relevant to malaria parasites in their human setting. Next, we use the trade-off theory to predict what the impact might be on the evolution of the pathogen's virulence if malaria vaccines went into widespread use. Finally, we summarise an experimental evolution study to test our prediction that enhanced immunity would select for more virulent parasites. Together, this work has led us to a deeper understanding of why malaria still kills its host despite millenia of coevolution, and what might happen when disease control campaigns change the level of population immunity, e.g., enhance it using vaccines, or reduce it using bednets and vector control.

### 1.1 The trade-off hypothesis and its assumptions

**Fig. 1:**
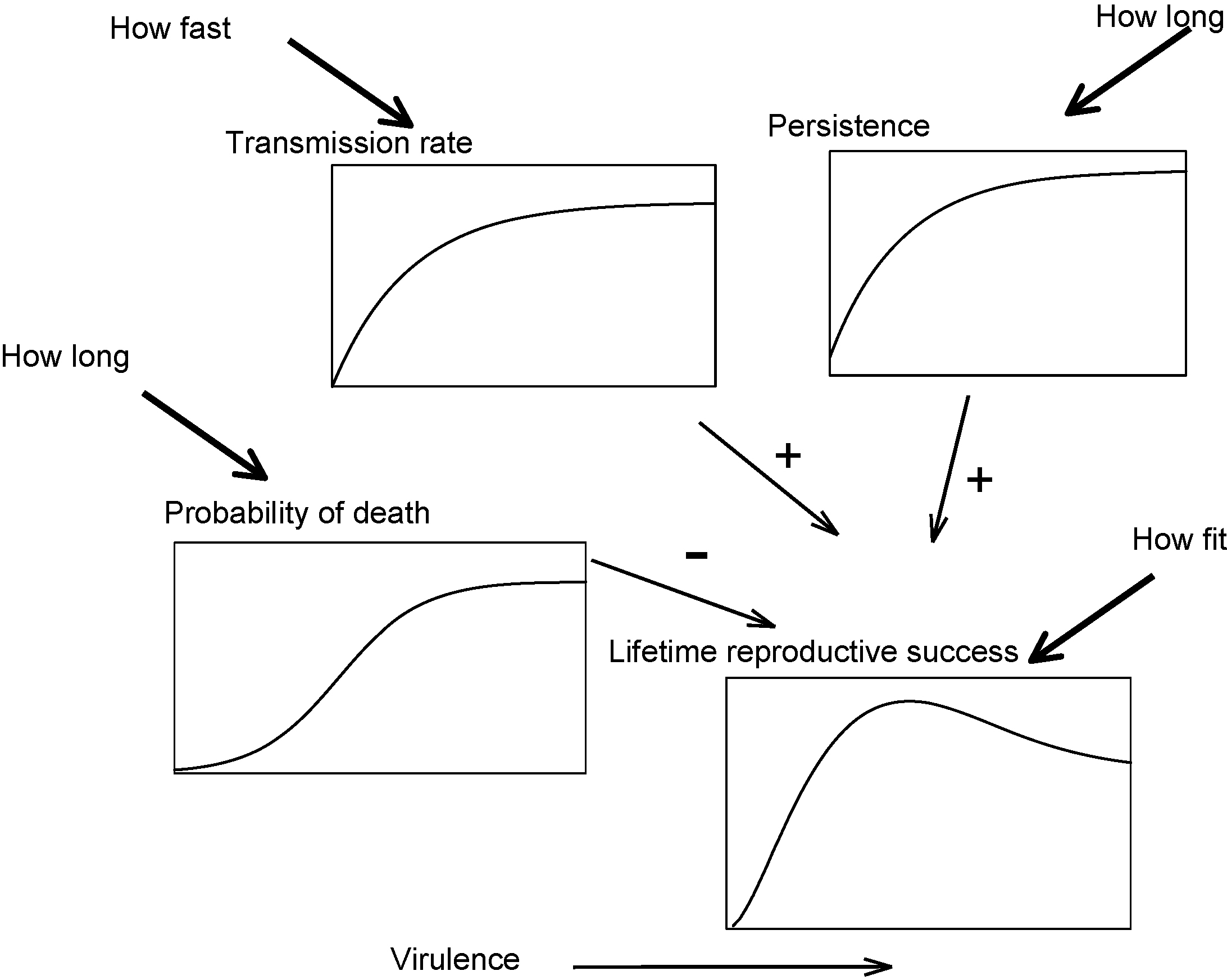
The costs and benefits of virulence (or, alternatively, high levels of host exploitation) to pathogen fitness. The fitness benefits associated with virulence are higher transmissibility and persistence (duration of infection). The fitness cost of virulence is host mortality which shortens the infection. The rate of transmission and length of infection (which is determined by both persistence and the probability that the host dies) multiply together to give the total lifetime transmission of the pathogen from the host, i.e., its fitness. This reaches a maximum at an intermediate level of virulence when the negative and positive effects on virulence are combined in this way. Note that the transmissibility and duration of infection curves have to be less than linear (i.e., convex) in order to produce an intermediate optimum virulence.

Under the trade-off hypothesis, it is assumed that there are both fitness benefits and costs associated with virulence. The cost is assumed to be host death because, for most pathogens, transmission stops when the host dies. The benefits associated with virulence are assumed to be production of more transmission forms per unit time, and/or increased persistence in a live host. However, the benefits of higher transmissibility and persistence only accrue if the host survives: if it dies, transmission immediately ceases, thereby directly reducing the pathogen's fitness. The pathogen is thus playing a perilous game, attempting to maximise transmissibility and infection length while also keeping its host alive. Pathogens with the highest fitness are those with an intermediate level of virulence which balances these opposing contributions to fitness (Fig. 1).

### 1.2 Evidence from myxomatosis

**Fig. 2:**
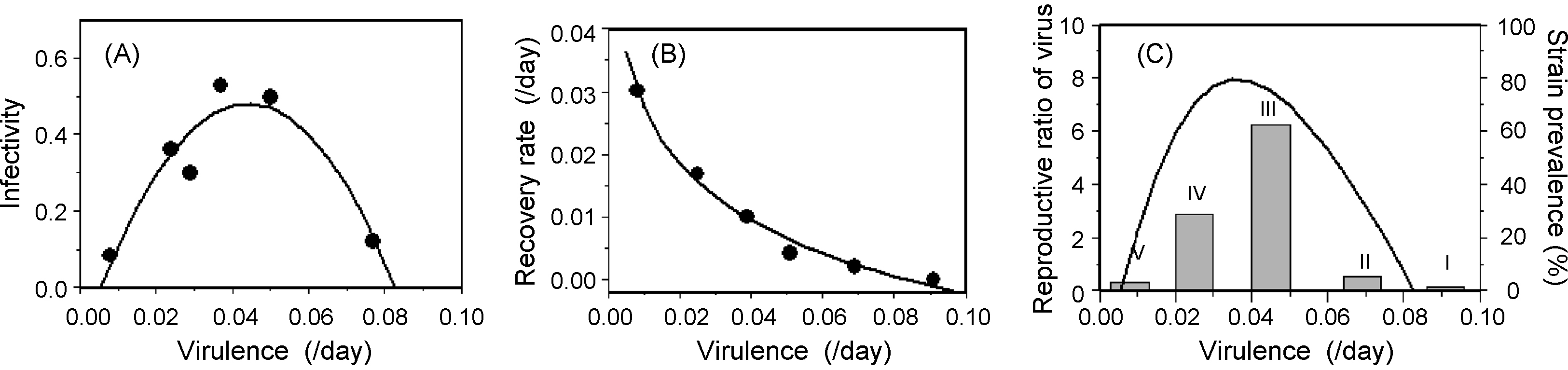
Virulence evolution in myxomatosis. A highly virulent myxomatosis virus was released into a rabbit population of Australia and Britain in the early 1950s. By the mid-1960s, strains of the virus that were less virulent than the original were found to be circulating in the populations (graded I–V in increasing order of virulence). The virulence of these strains showed positive, curvilinear relationships with infectivity to fleas/mosquitoes [12] (panel A) and the length of infection [11] (panel B) when measured in laboratory rabbits. Using either of these relationships, and keeping the other one constant, gives maximum fitness at an intermediate level of virulence [8], [13]: this is also the case when both vary, as shown in panel C. The predicted fitness function matches the distribution of strains observed in Australia after a decade (1959–1969) [80] where the most prevalent strains (grey bars) in the population were those of intermediate virulence, a situation which lasted for some time until the host evolved higher levels of resistance [81], [82]. Note that the very low infectivity of the highly virulent strain is not necessary to generate an intermediate virulence optimum: a transmissibility–virulence curve that saturated at 60% would too. Following Anderson and May [8], assumed values of other parameters were a background mortality rate of 0.011/day and values of the daily transmission rate to uninfected rabbits approximately equal to infectivity, i.e., 0.1–0.6 transmissions per infected rabbit per day.

Although the trade-off idea was mooted by medical epidemiologists early in the 20th century [5] and much later by theoretical biologists [6], [7], it was Anderson and May's [8] analysis of the principle using a real-life example which really focussed thinking. The myxoma virus was extremely virulent when released into rabbit populations in Australia and Britain in the early 1950s, but over the next decade, the virus evolved to an intermediate and stable level of virulence [9], [10]. Using data from Fenner and Ratcliffe [11] which showed that viral strains that caused more host death were also those capable of generating longer infections (Fig. 2a), Anderson and May [8] showed that maximum fitness of the virus occurred at an intermediate level of virulence. This was because the more virulent strains produced longer infections in the absence of host death, but death occurred more often in these strains. In their analysis they assumed that transmissibility was constant, i.e., the trade-off was generated exclusively by the relationship between virulence and infection length. Similarly, using the data of Mead-Briggs and Vaughan [12] which show higher transmissibility in the more virulent strains (except at very high levels, Fig. 2b), Massad [13] showed that maximum pathogen fitness was reached at intermediate levels of virulence. Thus either the virulence–infection length relationship or the virulence–transmissibility relationship, or the combination of the two (Fig. 2c) could explain the observed evolution of the myxoma virus from extreme virulence to intermediate virulence through time.

## 2 Malaria

Inspired by the myxomatosis example, and conscious of the lack of relevant data in medically important pathogens, we set out to explore whether this trade-off was operating in a major pathogen of humans—*Plasmodium*. The causative agent of the most pathogenic form of human malaria, *Plasmodium falciparum*, replicates rapidly within its host, as do many viral, bacterial, and other protozoal pathogens. Unlike viruses and bacteria, however, the malaria parasite has separate transmission stages called gametocytes. Gametocytes, the sexual form of the parasite, are differentiated from the asexual, replicating blood-stage forms. Gametocytes do not themselves replicate, and are seen at low densities in the bloodstream. But as they are the only forms that survive in the mosquito vector, the pathogen must produce them in order to be transmitted. Moreover, mosquitoes only feed on live hosts. In hosts that survive, immunity clears parasites. Thus, on the face of it, the necessary ingredients for the trade-off model are present: the most evolutionarily successful parasite is one which maximises transmission stage production while avoiding host death and immune clearance.

### 2.1 Data from mouse malaria

We began by testing the assumptions of the trade-off hypothesis using the mouse-malaria model, *Plasmodium chabaudi*, as an experimental model. Using parasite clones obtained from their natural host in the wild [14] and then passaged for a short period in laboratory mice, we infected groups of inbred mice and measured the transmissibility, virulence and persistence of these clones and the genetic (i.e., across-clone) relationships among these traits [15], [16], [17]. Transmissibility was measured as the number of gametocytes per unit time: it was further measured as the proportion of mosquitoes that became infected after being allowed to take a blood-meal from an infected mouse. Persistence was measured by the time it took to clear an infection to below a threshold value. Virulence was, for ethical and logistical reasons, measured not by mortality but by morbidity, viz., maximum level of anaemia or weight loss experienced by the host during the infection: these are positive indicators of the probability of host death. We also measured the maximum asexual parasite density reached during the infection as an indicator of the parasite's degree of exploitation of the host. In malaria, this is a useful trait through which virulence–transmission–persistence relationships (hereafter V–T–P) can be examined since parasite density is a major underlying cause of all three traits: asexual replication is directly responsible for disease pathology, production of gametocytes and for maintenance of the infection.

**Fig. 3:**
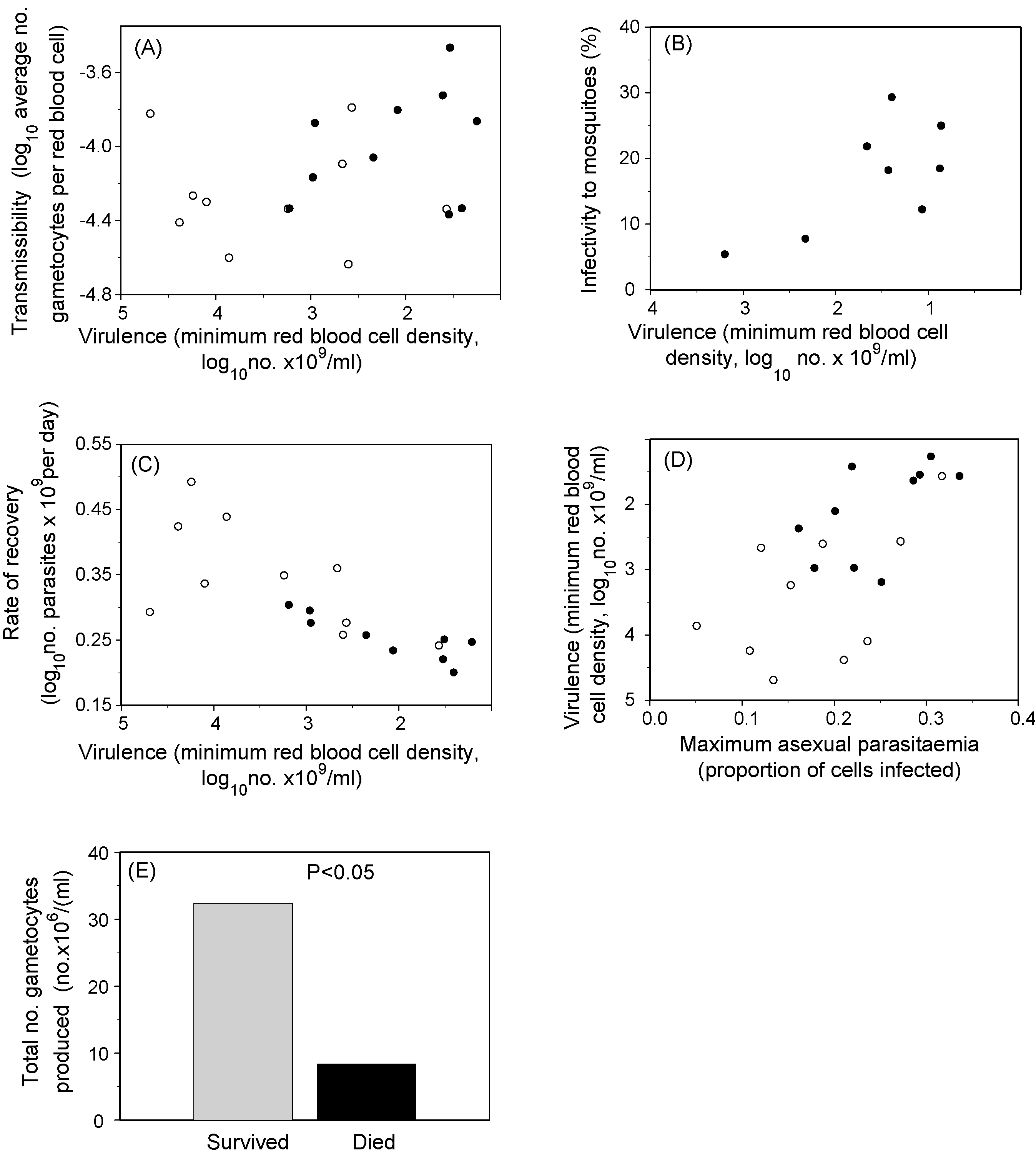
Virulence–transmissibility–persistence relationships in the mouse model of malaria, *P. chabaudi*. Each point is the average value of groups of mice (*n* = 5–10) infected with one parasite clone: thus a line fitted to the points would represent the parasite genetic relationship among the traits. Filled symbols indicate naïve mice and open symbols indicate mice made semi-immune by previous infection and drug clearance. Virulence was measured as the mouse's minimum red blood cell density reached during the infection. It was shown to positively relate to transmissibility as measured by daily average gametocyte density (panel A), and infectivity to mosquitoes on 2–4 days during the peak of gametocyte production (panel B). Recovery rate (panel C), which is inversely related to infection length, was measured as the rate at which the infection declined after peak parasitaemia and was negatively related to virulence, as expected. Virulence was positively related to maximum parasitaemia (panel D), an indicator of host exploitation. Clones broadly retained their rankings for all traits when infecting naïve vs. immunised mice (Pearson correlations across treatments of 0.59–0.66 for the four traits used here.) In the experiments described in panels A–D, very few mice died and so a virulence cost to transmission was not observable. However, in another experiment using less resistant host genotypes, mortality was high (23%) and was shown to severely reduce the total number of gametocytes produced by the infection, shown in panel E. Data were reproduced with permission from Mackinnon et al. [19] and Mackinnon and Read [16].

Our experiments showed that clones that had higher levels of virulence also had higher transmissibility (both production of gametocytes and infectivity to mosquitoes) and cleared their infections more slowly in the absence of host death (Fig. 3) [15], [16], [17], [18]. Virulent parasites were also those that generated highest parasite densities and thus exploited more of the host's resources (red cells). Thus the V–T–P relationships assumed by the trade-off hypothesis, and the parasite genetic basis for them, were supported in this experimental model. Furthermore, if the host died, the total amount of gametocytes produced during the infection (i.e., the potential transmission) was less than if the host lived (Fig. 3e) thus demonstrating a clear cost of host mortality to the parasite's fitness [19]. This result, however, was not repeated in a similar study [17] despite similar levels of mortality and gametocyte production in both experiments. Further and larger experiments are required to determine the magnitude of the cost of host death to parasite fitness. The findings described above were also broadly supported, at least at the phenotypic level, in another experimental system, the avian malaria, *Plasmodium gallinaceum* infecting chickens [20].

We found the V–T–P relationships also to be positive when measured in different host genotypes [19], [21], host sexes [22], after serial passage [18], [19], [22], after mosquito passage [22], [23] and, importantly (see below), in hosts with different levels of acquired immunity [16], [22]. Thus clones that were genetically more virulent, transmissible and persistent were consistently so, across a range of extrinsic, host-derived factors.

In nature, malaria infections are frequently composed of several genetically distinct strains of parasites. This introduces into the relatively simple scenario described above the question of whether the more virulent parasites are still more productive (i.e., fit) than their less virulent counterparts when they are competing in the same host. Theoreticians have envisaged a range of possible outcomes of this competition, and hence effects on virulence evolution (reviewed in [24]). Experimental data using the *P. chabaudi* model, generally indicate that more virulent clones have a competitive advantage: they are able to competitively suppress or even exclude less virulent clones during the acute phase of mixed-clone infections [25], [26]. The competitive outcome is reflected in relative gametocyte production of the two competing strains [27] and hence transmission to mosquitoes [25], [28]. This result appears to be qualitatively robust to host genotype [29] and host immune status [30], [31]. Thus, the transmission and persistence advantages of virulence accrue even in mixed infections.

Important to the argument that follows, in the mouse model, immunity acted to reduce all three traits – transmissibility, virulence, and persistence – in line with that expected from the relationships among these traits in naïve hosts. Also, clones broadly retained their rankings for virulence and other traits across naïve and immune hosts (Fig. 3). In other words, the parasite lines that replicated well, transmitted faster, lasted longer and caused more morbidity when infecting naïve hosts, also did so in semi-immune hosts but just at a lower level.

### 2.2 Data from human malaria

Do the V–T–P relationships we observed in mice apply to human malaria? Unfortunately there are not adequate data to properly assess this, and there probably never will be. This is because we cannot, for both logistical and ethical reasons, measure the virulence of a panel of distinct human malaria strains in groups of human hosts. However, two types of data which represent the phenotypic relationships among these traits are qualitatively consistent with the positive genetic relationships we observed in the laboratory mouse.

**Fig. 4:**
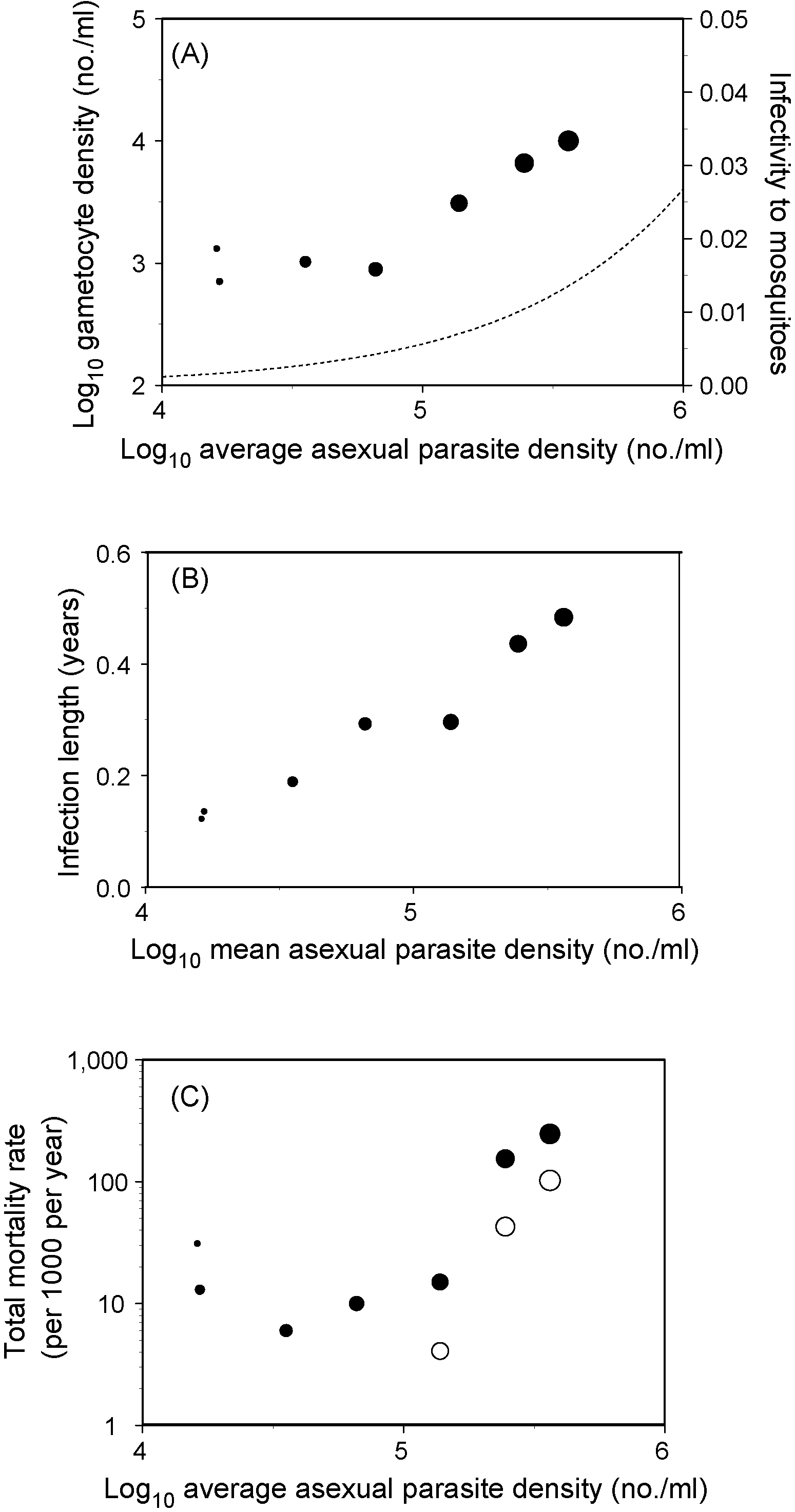
Relationships between level of host exploitation, transmissibility, persistence, and virulence (mortality) in human malaria in the field. Data were taken from a longitudinal study in Nigeria in the 1970s (the Garki project, [32]). Host exploitation is on the *x*-axis and is represented by average asexual parasite density. Each symbol represents an average for a group of people from the same age-group measured in eight surveys, and decreases in size with age. Transmissibility (panel A) is represented by gametocyte density, and then converted to infectivity (dotted line, right axis) using logistic regression fitted to data from experimental mosquito infectivity data from the literature (data not shown). Persistence (panel B) is represented by infection length estimated from consecutive 10-week surveys [83] and adjusted for superinfections to lengths of individual infections using the method described by Dietz et al. [84]. All-cause mortality rates (panel C) are shown as closed symbols: these are due to other factors as well as malaria, and include all the malaria infections that occur in a host per year, i.e., including superinfections. The open symbols represent the values of mortality assuming that 25% of all deaths under the age of 9 years, and 1% in older categories, are due to malaria: these values are representative of studies where these proportions have been estimated, albeit with considerable uncertainty [40].

The first type of data comes from a longitudinal cross-sectional population survey in Nigeria (the Garki project) [32]. The data from this are summarised in the form of plots of average values for each age-class of V, T and P (measured by mortality rates, average gametocyte density while infected, and duration of infection for each new infection, respectively) against average asexual densities, an indicator of the degree of host exploitation by the parasite (Fig. 4). Across the age-spectrum, all three traits increased with exploitation levels, consistent with those assumed by the trade-off hypothesis and observed in rodent and avian malarias.

However, two important caveats apply to these human malaria data regarding their relevance to the trade-off hypothesis. The first is that parasite genetics is averaged out in these relationships (each age-group is assumed to be infected by the whole range of parasite genotypes in the population). Second, the observed relationships are undoubtedly driven by age-acquired immunity. Thus these curves, tight as they are, do not directly yield the parasite's genetically encoded fitness benefits and costs of virulence as assumed by the model (Fig. 1), and seen in myxomatosis (Fig. 2) and rodent malaria (Fig. 3). However, the Garki data are certainly not inconsistent with the model.

A second source of data on V–T–P relationships is the series of infections of human patients with *P. falciparum* malaria in order to treat their neurosyphilis. Several laboratory-maintained strains were used to treat hundreds of patients, and their courses of asexual parasite densities, gametocyte densities, infection lengths, and morbidity (fever) were recorded for each infection for as long as 18 months, thus giving data relevant to V–T–P relationships [33], [34]. It was found that infections among malaria-naïve patients that reached higher maximum parasite densities, or had faster growth rates during the early stage of the infection, also persisted for longer, and caused higher levels of morbidity than infections that reached lower maximum densities (Mackinnon, unpublished results). However, as for the field studies described above, because few parasite strains were used, these relationships could not be attributed to parasite genetics. Thus we still do not have a basis on which to critically evaluate the assumptions of the trade-off model for human malaria.

It must also be noted, however, that there are various pieces of evidence to indicate the potential for virulence evolution in human malaria in response to changing selection pressures. These are as follows: there appears to be a parasite genetic basis to virulence, replication rate and perhaps transmissibility in *P. falciparum* (reviewed in [35]); there is some variability in virulence maintained in nature [36], [37]; and there appears to be a link between intrinsic replication rate and virulence in *P. falciparum* [38] (but see [39]). We recognise that there are many environmental and genetic factors relating to both the vertebrate and mosquito hosts that contribute much variability to the V–T–P relationships in human malaria. What matters, however, to the direction of virulence evolution is whether, when averaged over all this variability, the parasite-encoded V–T–P relationships are qualitatively similar to that assumed by the theory (Fig. 1).

Our experimental work with *P. chabaudi* clearly demonstrates fitness benefits to virulence (transmissibility, persistence, and competitive ability). These are thus factors that promote the evolution of increased virulence. What factors balance these, preventing the evolution of extremely virulent malaria? The trade-off model envisages the balancing selective pressure to arise through host death. But in the field, the majority of malaria infections do not end in death. Determining what the case fatality rate is for malaria in endemic regions is difficult, so that estimates are controversial, but the probability of a single infection leading to death is certainly less than 10% and probably less than 1%, perhaps even <0.1% [40]. Is such a low risk of death a sufficient cost of virulence to offset the fitness benefits we have seen? (Note that even a small amount of mortality may be sufficient to counterbalance a fairly flat transmissibility curve.) To work this out definitively, we would need to determine both the transmission benefits and transmission costs associated with higher death rates. The population-wide case fatality rate is not a good indicator of whether evolution of high virulence is being constrained by host death because it does not directly reflect these transmission gains and losses. We would also need to take account of the variable amount of transmission from hosts with different levels of immunity. Thus the optimum virulence at endemic equilibrium will not only depend on the shape of the virulence–fitness curve, but also on the parasite's productivity (total transmission per infection lifetime) in each host type, and the abundance of each host type [41], [42], [43], [44], [45].

**Fig. 5:**
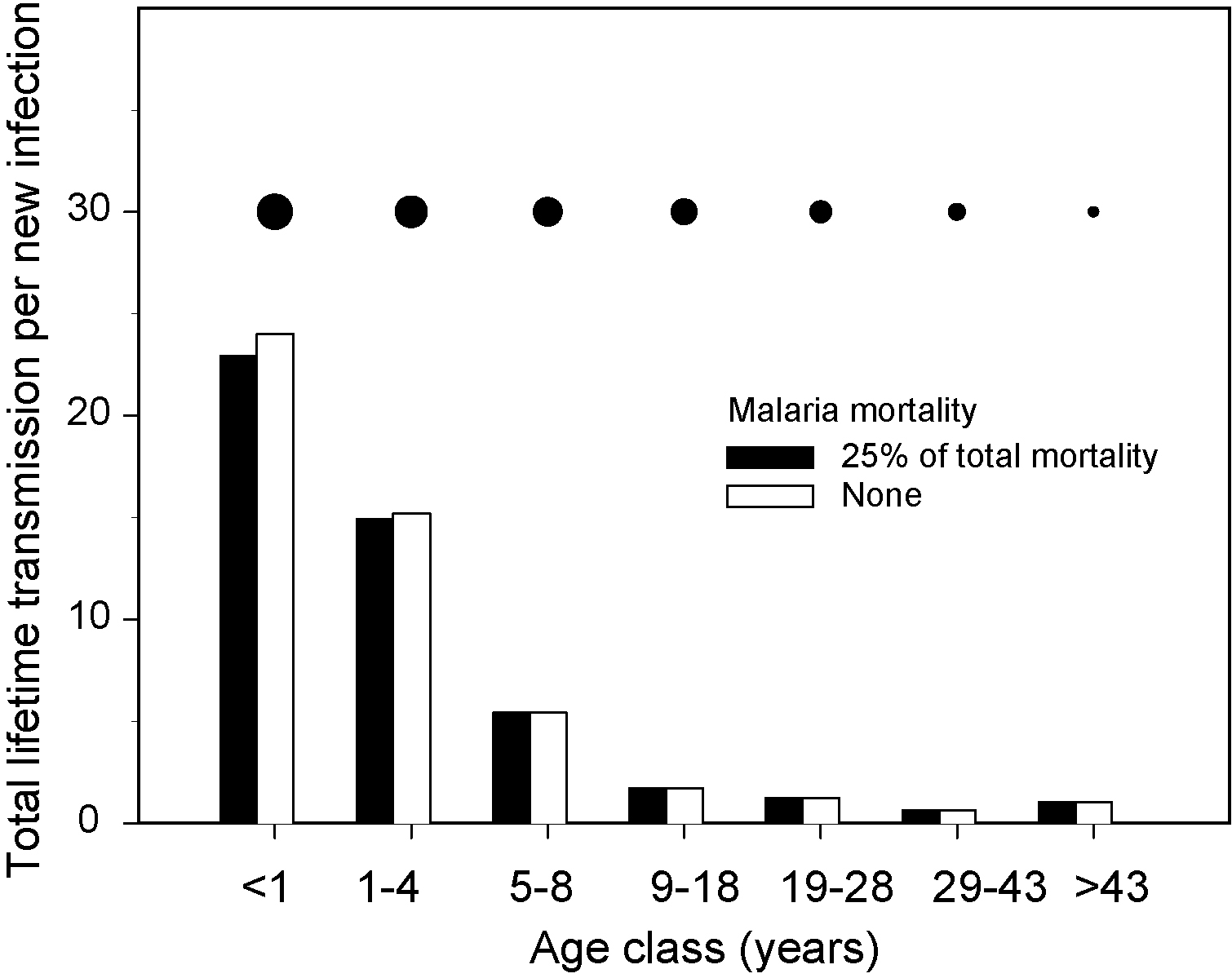
The expected amount of total transmission from hosts in different age-classes in the Garki project based on the data shown in Fig. 4 gives some indication of the parasite's relative fitness in different host types. Total transmission (black bars) was calculated as infectivity multiplied by vectorial capacity (the expected number of infectious bites to new hosts that result from a mosquito feeding on one host, infected or not, assumed to have a value of 8 here) divided by the expected infection length. Infection length here is the reciprocal of all-cause mortality rate (Fig. 4c) plus recovery rate (the inverse of individual infection length, Fig. 4b). We use the length of an individual parasite infection in this calculation because we are interested in the relative transmission, or fitness, of an individual parasite strain rather than the total transmission from a group of coinfecting strains occupying the same host. By contrast, mortality rate in this calculation is all-cause mortality because individual parasite genotypes suffer from the mortality caused by coinfecting parasites as well as that caused by themselves. To illustrate the cost of host death on the parasite's total lifetime productivity, we have performed the same calculation as above but assuming that none of the all-cause mortality is due to malaria (see Fig. 4 legend) (white bars). The different-sized symbols above the bars correspond to those for the different age-classes in Fig. 4.

Data from the Garki project help to illustrate this last point. Mortality rates are much higher in children, especially very young children, where they are two orders of magnitude higher than in adults (Fig. 4c). But it is also these younger (non-immune) groups that have the capacity to generate high gametocyte densities and persistent infections (Fig. 4a and b). As Fig. 5 shows, this is the group that contributes most (20-fold more than adults) to the parasite's total transmission population, but also suffers the highest virulence and hence cost to transmission. Thus one might plausibly argue that it is the youngest children, who transmit most but die most often, that have the most ‘weight’ in determining the optimum level of virulence for the population. Moreover, more virulent parasites could be maintained in the population by semi-immune, transmitting adults, because they are less likely to kill their hosts. Thus if looked at from the host's point of view, one could say that the young children are paying the cost of adult immunity. This raises the question we now turn to: how will virulence evolution be altered when a greater proportion of the parasites are facing immune hosts, as would occur following widespread vaccination?

## 3 The vaccination theory

### 3.1 The principle

Before going on to describe the principle of our vaccination theory, we define two terms which will be used in the following discussion: these are *intrinsic* and *realised* virulence. Realised virulence is the virulence that would be observed in a host at a given level (any level) of host defence. Intrinsic virulence is the virulence that would be observed if the parasite was infecting a naïve host: in a better-defended host, for the same level of intrinsic virulence, the realised virulence would be lower. We need intrinsic virulence as a common reference in order to study changes in virulence in populations of hosts with mixed levels of defence.

**Fig. 6:**
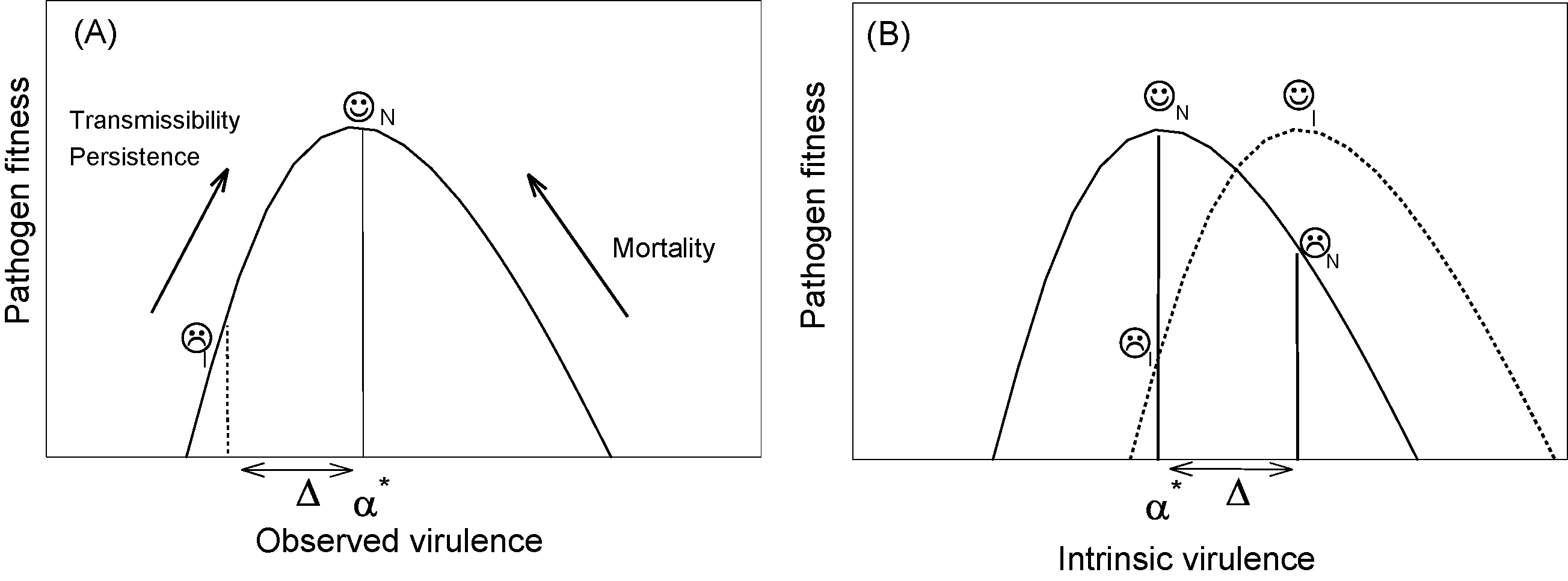
Immunity selects for higher levels of intrinsic virulence. In panel A, the solid line shows the fitness curve for parasites as a function of observed (or realised) virulence in naïve hosts. In naïve hosts, it is maximised at some intermediate virulence level, α^*^, as indicated by the  symbol. However, if the parasite finds itself in a semi-immune host, its fitness is lower than maximal (vertical dotted line) because of its lower (by amount *Δ*) or observed virulence, hence the  symbol. In panel B, the fitness is shown as a function of *intrinsic* virulence, i.e., that which would be observed in naïve hosts. In naïve hosts (solid line), α^*^ is the optimal level of virulence, as in panel A. However, in semi-immune hosts (dotted line), the optimal level of intrinsic virulence fitness would be maximised at a higher level of virulence, viz. α^*^ + *Δ.* This is because its realised virulence would be this value, less the effect of immunity, *Δ*. But this optimal level of intrinsic virulence at α^*^ + *Δ* would be too high for a naïve host (solid line), as indicated by the  symbol.

If there are intrinsic biological links between the V–T–P traits that cover the whole spectrum of host defence, the parasite in semi-immune hosts is subject to the same fitness trade-off as proposed for naïve hosts (Fig. 1). The only difference is that the realised cost of virulence, host death, is lower in immune hosts than the realised cost in naïve hosts. This means that in semi-immune hosts, the parasite, in order to achieve higher effective replication rate, transmission and persistence, can afford a higher level of *intrinsic* virulence without paying the cost of host death. In other words, parasites with high virulence can ‘get away with it’ in a semi-immune host. Therefore intrinsic virulence should evolve to be higher in populations with higher levels of immunity than in populations of naïve hosts [4], [43], [46], [47], [48], [49], [50]. In our model the amount by which it does so in a population consisting entirely of immune hosts, relative to that for a completely naïve population, is the exact same amount by which immunity decreases realised virulence over intrinsic virulence. This principle is illustrated in Fig. 6. Note that under the assumptions of our model, this principle would apply to any pathogen where the trade-off holds, and for any form of host resistance (genetically innate, naturally acquired, or vaccine-induced) that operates to reduce parasite virulence, e.g., via reduced parasite growth rates or anti-toxin effects [43], [51].

### 3.2 Application to asexual-stage vaccines in malaria

The data from mice and humans strongly indicate that in malaria the common factor underlying the V–T–P relationships is asexual parasite density. We reasoned, therefore, that any control measures that brought about a reduction in asexual parasite densities would bring about evolution for increased intrinsic virulence because of its consequences to transmissibility, persistence and host mortality [43]. This would include anti-asexual-stage vaccines and drugs which were imperfect or ‘leaky’, i.e., that did not completely prevent or clear infections, respectively. If this evolution were to happen, the unprotected (e.g., unvaccinated) people in the population would then be exposed to a more virulent parasite and their risk of death would be higher than before.

On the other hand, it might be argued, the vaccine, if used widely, would protect many more people from disease so that the population-wide reduction in mortality may well outweigh the increased mortality among the unvaccinated few. Further, the vaccine would reduce the force of infection through its effect on transmissibility, thus conferring an extra benefit to the population through herd immunity [52]. This would further relieve the natural selection pressure on the parasite's virulence from naturally acquired immunity. It would also reduce the incidence of multiple infections: all of these effects are generally considered to be beneficial. Thus, as is often the case in vaccination programmes, even without evolution, the benefits to the majority of the population have to be weighed against the risk to a few individuals.

**Fig. 7:**
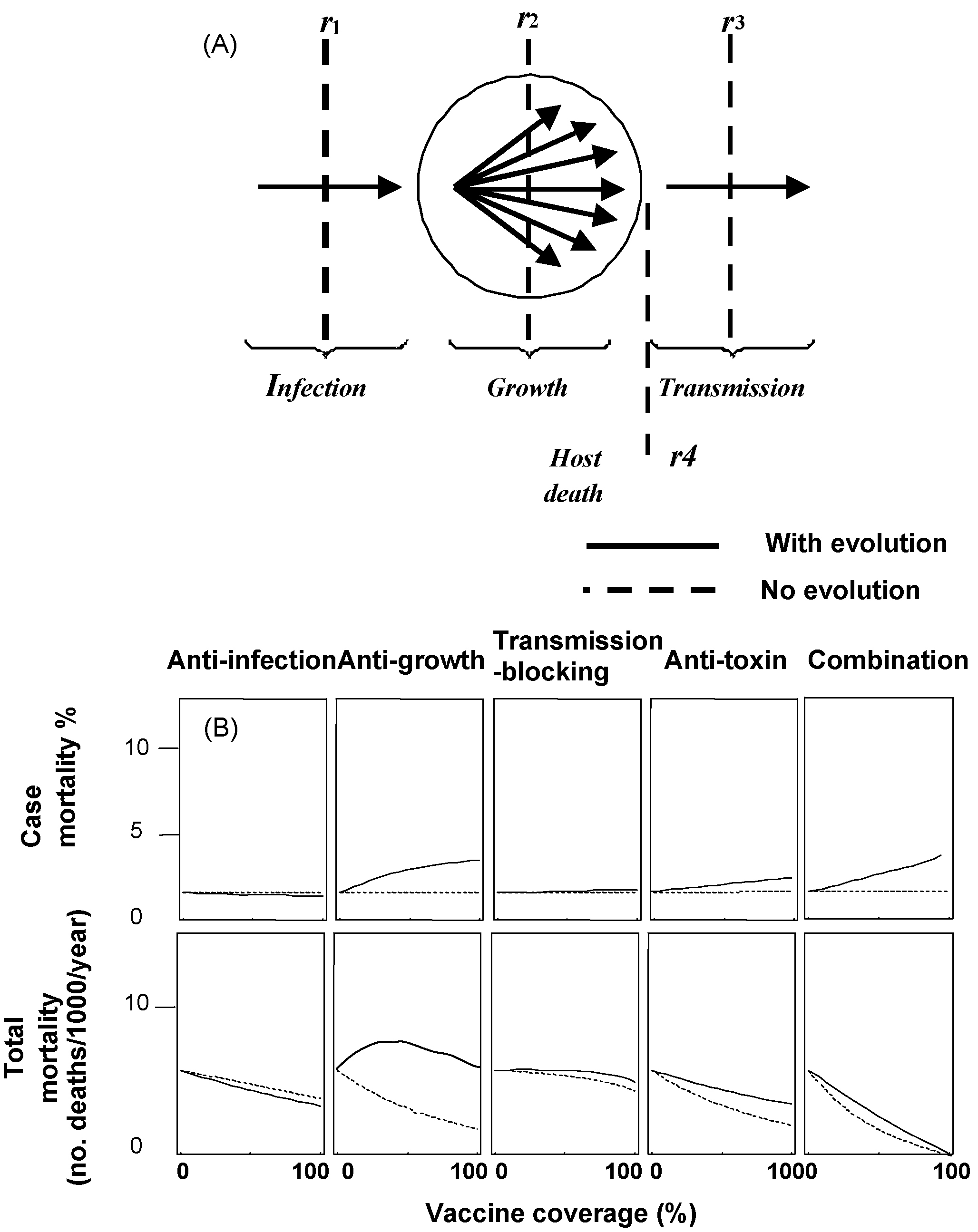
Vaccines (or other control devices) can be targeted at different stages of the malaria parasite's life cycle (panel A). r_1_-type devices block infection (e.g., a liver-stage vaccine), r_2_-type devices act against replication (e.g., asexual-stage vaccines), r_3_-type devices act against transmission from the host (e.g., transmission-blocking vaccines or bednets), and r_4_-type devices act directly against the toxicity of the pathogen (e.g., anti-toxin vaccine, or life-saving therapies). The consequences of virulence evolution to unvaccinated hosts (top panel) and the total population of hosts (vaccinated, unvaccinated and naturally immune) are shown as a function of vaccine coverage (*x*-axis) (panel B). The solid lines show the mortality expected if the pathogen evolves, and the dotted lines show the consequences if it does not. For an r_2_-type vaccine, virulence evolution is expected to erode all the benefits of vaccination and cause more death in unvaccinated individuals. In contrast, r_1_-type and r_3_-type devices are expected to select for avirulence. Preventing death using an r_4_-type device selects for higher virulence in unvaccinated people, but is beneficial to the overall population. The effect of a combination vaccine is shown in the last column. Reproduced with permission from Gandon et al. [43].

Therefore, to determine the benefit to the whole population of vaccination allowing for an evolutionary response in the parasite's virulence, we modelled the case of virulence evolution under an imperfect asexual-stage malaria vaccine incorporating the feedbacks between the epidemiology (force of infection) and parasite evolution [43]. Parameter values were chosen to mimic an area of high year-round malaria transmission such as Tanzania. As predicted, the parasite evolved higher virulence under vaccination so that the case fatality rate amongst unvaccinated naïve hosts was higher than if evolution had not occurred. Moreover, the total mortality across the whole population was also higher, especially when the vaccine was given to a moderate fraction of the population. Thus all the expected benefits of vaccination were eroded by parasite evolution, and the situation was worse at intermediate levels of vaccine coverage (Fig. 7).

### 3.3 Other types of vaccines and coinfection

We also modelled other types of vaccines or devices that block infection (e.g., liver stage vaccines), or stop it transmitting from humans to mosquitoes, (e.g., bednets), but that do not act directly on the asexual-stage parasites and hence on the V–T–P relationships. These control measures were predicted to have no impact, or as we explain below, could even lead to a decrease in virulence [43]. Thus, priority should be given to control measures that prevent pathogens from entering or leaving the host rather than measures that directly target the infection itself. In malaria, the former would include bednets, vector control, and sporozoite and liver stage vaccines (unless they also reduce multiplication rates); the latter includes asexual-stage vaccines, anti-toxin vaccines and drug use. Thus, in the face of a large potential for pathogen evolution, where possible, to control the disease, the hygiene principle would appear to be an excellent one to follow for evolutionary reasons as well as for its more obvious epidemiological applications.

The theoretical conclusion that using devices that prevent infection or block transmission will lead to reduced virulence arises because of the lower force of infection (transmission intensity from the vector population) caused by the control programme. This leads to lower levels of coinfection or superinfection and hence the lower level of competition among parasites occupying the same host. The reduced selection pressure for high virulence due to relaxation of competition can be understood by taking the view of a resident parasite that is either ousted by (superinfection) or forced to share with (coinfection) another parasite type. In the case of superinfection, the resident parasite has its infection shortened by the invading parasite: the parasite then compensates by evolving a higher level of host exploitation, or intrinsic virulence, up to the point when the benefits are balanced by the costs. This is especially so where virulence is associated with competitive ability, as it is at least in *P. chabaudi* (see Section 2.1). In the case of coinfection, the risk of mortality for the resident parasite is increased by the presence of another parasite, thus increasing the costs. Again, the parasite has to compensate by increasing its level of host exploitation (benefits).

The principles of superinfection and coinfection also extend to co-morbidity from other infections. In malaria, concomitant infection with bacterial pathogens greatly enhances the probability of dying in young children [53]. This higher ‘background mortality’ in children is generally expected to heighten the selection pressure on virulence [54], [55] (but see [56]). Thus controlling other diseases may also relieve the selection pressure on malaria virulence. Indeed, as discussed above, a general principle that arises from the trade-off model is that if external factors act to decrease the duration of infection, then optimum virulence increases. These factors may be related to host death (e.g., war, co-morbidity, and mosquito mortality), or to the rate of clearance of the infection (e.g., superinfection, or drugs).

## 4 An experimental test of the vaccination theory

**Fig. 8:**
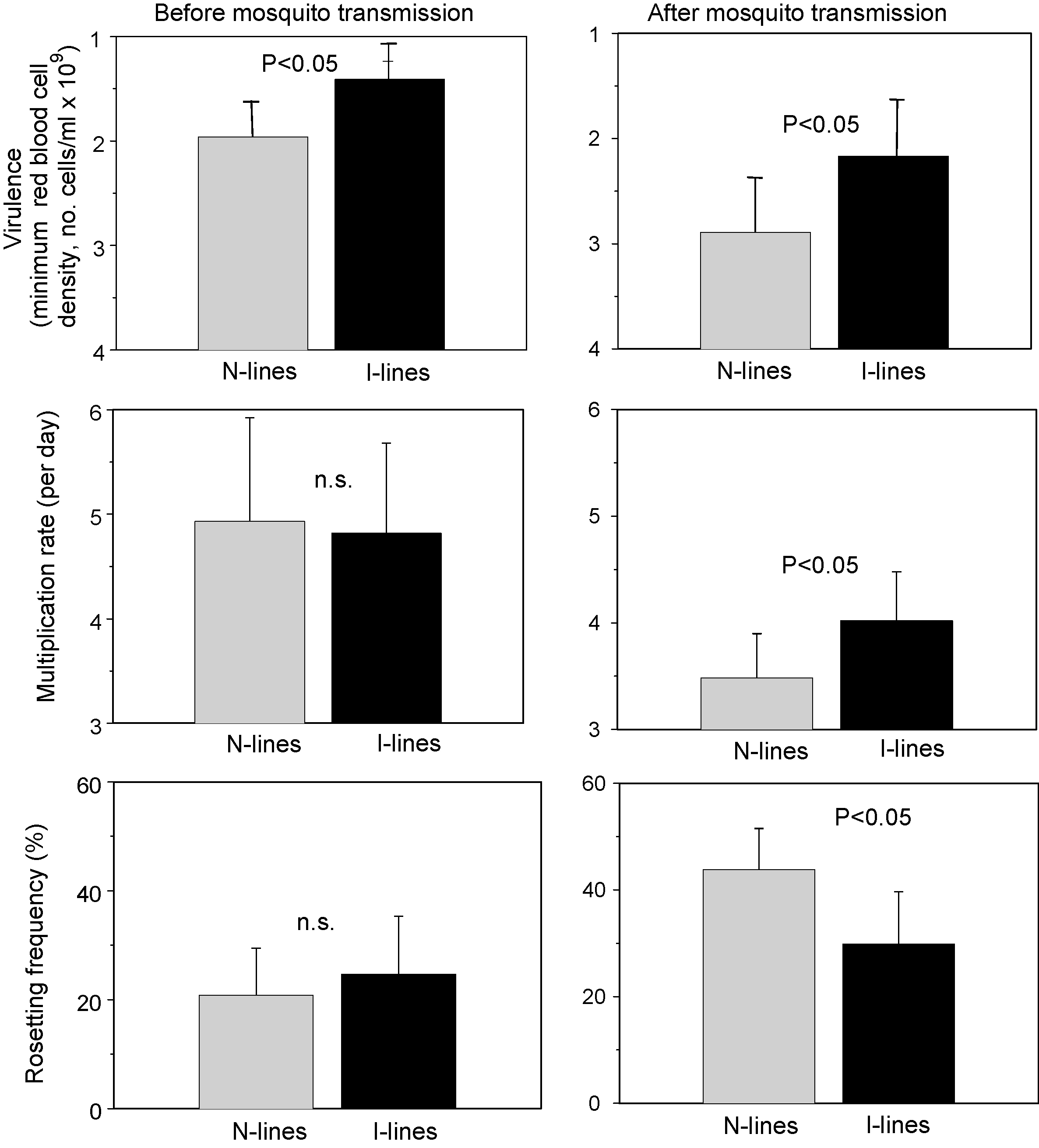
Results of serial passage of *P. chabaudi* through either immunised (I-lines) vs. naïve (N-lines) mice. Starting with an avirulent clone, five independent parasite lines were blood-passaged in mice every 7 days for 18 passages (see [22], for further details of the experimental design). At the end, the lines were passaged through mosquitoes once, and then compared for their virulence (measured by the minimum blood cell density reached during the infection), the rate of population growth during the first 6 days using real-time PCR (see [23] for details of methods), and rosetting frequency (the proportion of infected red blood cells that bind to more than two uninfected cells thus forming clusters, and the only molecular phenotype in human malaria that is consistently associated with virulence [85], [86], [87], [88]). Heights of bars represent means (with 95% confidence intervals) of groups of mice infected with the five selection lines per selection treatment. Selection in immunised mice generated more virulent parasites than in naïve mice indicating that immunity selects more intensively for virulent forms during within-host selection. These differences were present in the lines both before (graphs on left) and after (right) mosquito transmission indicating that at least some of the evolved difference was genetically stable. These parasites also multiplied faster during the early, non-growth-limiting stage of the infection before the onset of disease and a strong immune response. This difference was not apparent in the lines before mosquito transmission, probably due to the maximum growth potential (around fivefold per day [23]) having been reached. Rosetting was also the same in the two sets of lines before mosquito transmission but different after transmission, and in the opposite direction to that expected from human malaria, i.e., it was lower in the most virulent lines. Differences between pre-and post-mosquito transmission were significant for all three traits (virulence, *P* < 0.05; multiplication rate, *P* < 0.001; rosetting, *P* < 0.05). Data in the top two figures are reproduced with permission from Mackinnon and Read [22].

As we cannot test the vaccination hypothesis in the field until it is too late, we would ideally test it in the laboratory in mice or *in vitro*. This could be done by evolving replicate parasite lines under conditions of high vs. low immunity using their natural transmission system. However, it is extremely difficult in the laboratory to repeatedly transmit multiple parasite lines of malaria through mosquitoes for the entire period of their transmissibility: it is also ethically challenging to allow mouse death to act as the selecting agent. Therefore we designed an experiment to test one component of the vaccine hypothesis, namely, that immunity selects for higher rates of host exploitation (regardless of the transmission consequences). This was done by transferring a standard number of parasites between mice every 7 days for 18 passages, thus bypassing the mosquito stage of the life cycle. At the end of this selection phase, the replicated lines were passaged once through mosquitoes and tested for virulence and other characteristics in either naïve or semi-immune mice. We found that parasite lines that had evolved in immunised hosts were more virulent than parasite lines that had evolved in naïve hosts (Fig. 8). This was true whether the lines were infecting naïve or immunised mice, and whether or not the lines had been passaged through mosquitoes prior to testing them [22]. The immune-selected lines also had higher intrinsic replication rates than the naïve-selected lines (Fig. 8). Thus immunity had more efficiently selected the parasites with greater ability to exploit the host and thus cause more virulence. This result is consistent with our theory that blood-stage vaccines would select for more virulent parasites, but the mechanism of selection in this experiment was different to that we proposed for human malaria in the field because we did not allow the mortality-induced transmission costs to do the selecting. The results from this experiment nevertheless support the underlying basis of the trade-off model, namely, that replication rate is the parasite's key to transmission success, especially in semi-immune hosts, with virulence being an unfortunate side-effect.

It will be impossible to do a direct test of the vaccination hypothesis in human malaria before the uncontrolled experiment is done on a vast scale. But there are two approaches that could be taken in the meantime. First, one could examine the outcome of the natural experiment that has been done. According to the theory, parasites from highly immune populations (naturally acquired) should be intrinsically more virulent than parasites from less immune populations. Thus a comparison of case fatality rates and disease severity among naïve individuals could be made from high transmission intensity areas (where population immunity is high) and from low transmission areas. There is some limited evidence to support this argument [57], [58], but see Reyburn et al. [59]. The hypothesis could also be tested by comparing mortality rates of malaria-naïve travellers who have acquired their infection from high vs. low transmission areas. A second way would be to compare *in vitro* the virulence-related molecular phenotypes of parasites from areas with contrasting transmission intensities. Studies taking both approaches are under way.

## 5 Vaccine-driven virulence evolution, and other diseases

One of the few large-scale malaria vaccine trials to date has already demonstrated vaccine-driven evolution [60]. The Combination B asexual blood-stage vaccine consists of several blood-stage antigens. During a phase II trial in Papua New Guinea, it was shown that vaccination reduced the prevalence of the allele of one of the antigens used in the vaccine. The alternative allele, which was not present in the vaccine and which rose in frequency, had in previous studies been associated with more virulent infections [61].

In principle, a variety of different types of mutants could evolve in response to vaccination [62]. These could include antigenic variants that are less readily recognised by vaccine-induced immunity thus giving rise to ‘serotype replacement’ [63], [64], [65]. However, there is no *a priori* reason to think such antigenic escape variants will have lower or higher intrinsic virulence than the one targeted by the vaccine. In this paper, however, we have argued that there exist variants that have a fitness advantage in semi-immune hosts independent of antigenic type, e.g., due to higher intrinsic growth rate. In the absence of vaccination, these variants are selected against because of their excessive virulence in naïve hosts, but are favoured in vaccinated populations where they are protected from the fitness costs of host death. Thus such variants could have changes at antigen loci, and these may or may not affect replication rate, but they could also include loci other than those encoding antigens, such as genes encoding replication rate, toxin production, and immunosuppressive compounds, and these have the potential to confer great virulence. For malaria, we do not have a clear idea which are the virulence genes, whether they confer intrinsically higher virulence or just immune escape and therefore what the effects a particular vaccine might have on virulence evolution. But our arguments above, combined with our experimental evolution results (Fig. 8) and the experience of the combination B vaccine [60] suggest that whatever the mechanism, we should consider in advance the possible evolutionary outcomes of imperfect interventions.

Are there any examples in other pathogens where vaccines have driven the pathogen towards higher virulence? Vaccines against smallpox, measles and polio have been outstandingly successful. These vaccines induce near-sterilising immunity. On the other hand, for diseases where vaccines are leaky, so that wild-type pathogens transmit and thus evolve through immune people, changes in pathogen virulence following vaccination have been documented, such as diphtheria, pneumococcal disease and whooping cough (pertussis). These cases have been discussed in more detail elsewhere ([62], [66]; Gandon and Day, this issue). While these examples demonstrate that pathogen evolution in response to vaccines can happen, the effects on virulence are extremely difficult to determine because comparisons of virulence are very difficult without contemporaneous experimental infections with the different pathogen strains. For many animal diseases, where such experiments can be done, successful vaccines are frequently leaky. There are clear examples from the poultry industry of substantial increases in virulence following the widespread introduction of vaccines to both Marek's disease and Infectious Bursal Disease (reviewed in [66]). These cases have all the hallmarks of vaccine-driven generalised virulence evolution. However, experimental analysis of their V–T–P relationships is still required to determine whether vaccination was indeed the cause. The subsequent history of the myxoma virus evolution demonstrates the role host resistance can also play in driving virulence evolution. As wild rabbits evolved greater resistance to myxomatosis, the virus evolved greater virulence (reviewed by [66], [67]).

Many new generation vaccines against human diseases are, like candidate malaria vaccines, very likely to be substantially leaky because natural immunity against their causative pathogens is far from perfect, or the pathogen has good mechanisms for immune evasion or suppression (e.g., HIV, Hepatitis B and C). In diseases for which the V–T–P relationships are as they are in myxomatosis (Fig. 2) and rodent malaria (Fig. 3), we expect our argument to apply wherever imperfect vaccines are in widespread use. For diseases where the V–T–P relationships are qualitatively different to those described above, vaccines could produce other evolutionary outcomes (e.g., [51]).

The current situation is, however, that for most human diseases we simply do not know the V–T–P relationships: indeed, in our view, much of the controversy surrounding the trade-off model [68], [69], [70], [71], [72], [73] arises because of the lack of direct experimentation on medically relevant diseases. In the molecular era, it is now feasible for many disease systems to properly quantify pathogen transmission and persistence for individual infections for the range of circulating pathogen genotypes. Because of the variability in virulence and transmissibility during the infection, this is ideally done for the full course of the infection. For example, of key importance is the time at which host mortality occurs in relation to when the bulk of transmission occurs. If death occurs early in the infection (e.g., in malaria where transmission does not occur until after the most dangerous point in the infection), then loss of transmission is high and so the cost relative to the benefit is high: if it occurs late (e.g., in HIV or tuberculosis), much of the transmission has already occurred prior to host death, and the costs are relatively lower [74]. Thus these pathogens can “afford” higher mortality rates. Such considerations, and others such as the role of immunity, and other in-host limitations in generating the trade-off curve [44], [49], [75], [76], [77], [78], [79], highlight the importance of understanding within-host dynamics to properly evaluate the V–T–P relationships in both acute and chronic infections. This is an area we have so far paid little attention to in malaria, but we believe is one that is likely to be useful for properly quantifying the fitness benefits and costs of virulence in human malaria, and hence evaluating the applicability of the trade-off model to this disease.

## 6 Conclusions

In the case of malaria, for which a vaccine is not yet available but is being intensively pursued, we have presented the case for why virulence may evolve in response to vaccines, what we might do to avoid it, and what we further need to do to determine its likelihood. Key points are:

• In rodent malaria, virulence, transmissibility and persistence appear to be intrinsically linked through the parasite's biology/life history, and these links are maintained over different levels of host immunity. We are unaware of any data on human malaria which contradicts this picture, though field and clinical data are necessarily more ambiguous. Immunity has a very strong impact on parasite fitness through its effect on reducing all three V–T–P traits in accordance with their biological links, and by reducing host death and hence the fitness cost of virulence.
• Our simple models, informed and parameterised from experimental work on rodent malaria and from human field data, predict that interventions that reduce asexual density but do not block infection, such as anti-asexual-stage vaccines, and drugs, are expected to lead to evolution of higher virulence, while hygiene (i.e., preventing infection and transmission) is expected to lead to evolution of lower virulence.
• More data are required in human malaria to determine whether the V–T–P relationships are encoded by parasite genes, and to determine how much virulence variation exists in the field. Tests of the immunity-virulence evolution hypothesis in human malaria, where possible, are urgently required. Better knowledge of the contributions to transmission by adults and children would help identify the most important source of selection pressure and hence help direct age-targeted interventions.
• Meanwhile, a cautionary approach to the widespread use of anti-replication or anti-disease vaccines seems justified. Ideally, this means combining such vaccines with transmission-blocking vaccines, bednets, drugs, housing improvements and other transmission-reducing measures. If a plurality of approaches is not possible, we urge long term monitoring of virulence trends, and the continual development of alternative anti-malarial measures. The history of malaria control is one of continual failure in the face of evolution.

## Footnotes

1 Throughout this paper, we strictly define virulence as the fitness cost that the parasite causes the host. This may be through mortality, or morbidity-related reduction in fertility or fecundity. We sometimes use morbidity as a surrogate measure of virulence.
